# Pharmacokinetic-Pharmacodynamic modelling of intracellular *Mycobacterium tuberculosis* growth and kill rates is predictive of clinical treatment duration

**DOI:** 10.1038/s41598-017-00529-6

**Published:** 2017-03-29

**Authors:** Ghaith Aljayyoussi, Victoria A. Jenkins, Raman Sharma, Alison Ardrey, Samantha Donnellan, Stephen A. Ward, Giancarlo A. Biagini

**Affiliations:** Research Centre for Drugs and Diagnostics, Liverpool School of Tropical Medicine, Liverpool, L3 5QA UK

## Abstract

Tuberculosis (TB) treatment is long and complex, typically involving a combination of drugs taken for 6 months. Improved drug regimens to shorten and simplify treatment are urgently required, however a major challenge to TB drug development is the lack of predictive pre-clinical tools. To address this deficiency, we have adopted a new high-content imaging-based approach capable of defining the killing kinetics of first line anti-TB drugs against intracellular *Mycobacterium tuberculosis* (*Mtb*) residing inside macrophages. Through use of this pharmacokinetic-pharmacodynamic (PK-PD) approach we demonstrate that the killing dynamics of the intracellular *Mtb* sub-population is critical to predicting clinical TB treatment duration. Integrated modelling of intracellular *Mtb* killing alongside conventional extracellular *Mtb* killing data, generates the biphasic responses typical of those described clinically. Our model supports the hypothesis that the use of higher doses of rifampicin (35 mg/kg) will significantly reduce treatment duration. Our described PK-PD approach offers a much needed decision making tool for the identification and prioritisation of new therapies which have the potential to reduce TB treatment duration.

## Introduction

Tuberculosis (TB) is a highly infectious disease, with one third of the world’s population being latently infected and accounting for an estimated 1.5 million deaths in 2014^1^. It is widely acknowledged that in the era of the Sustainable Development Goals, continued reduction in TB-related deaths will require improved tools for TB control, among them more effective drug regimens capable of shortening and simplifying treatment of the disease from months to weeks, and drugs that are effective against multi drug resistant (MDR) and extensively drug resistant (XDR) strains which can make the disease untreatable. A major challenge to TB drug development is the lack of validated predictive *in vitro* and *in vivo* pre-clinical tools that can be used to identify potential new drug candidates or drug combinations/regimens that confidently translate to shorter treatments in the clinic^2, 3^. In the case of moxifloxacin (MOX) for example, efficacy data acquired using current *in vitro* and *in vivo* models predicted the potential for a shortened treatment regimen^4–6^. Unfortunately, these pre-clinical model end points did not translate to humans and in a recent clinical trial in which MOX replaced either ethambutol (EMB) or isoniazid (INH), MOX did not shorten the standard 6 month TB treatment to 4 months^7^. One conclusion from this costly and time-consuming exercise is that we currently do not have validated pre-clinical discovery strategies capable of confidently supporting decision-making.

Typically, clinical studies report a biphasic response in the bacillary load to treatment with short-course regimens (e.g. ref. 8). A long-standing explanation for this phenomenon, is that the target population of *Mycobacterium tuberculosis* (*Mtb*) in patients is heterogeneous in terms of its response to different components of therapy^9^. Generally, it is accepted that in order to understand the overall pattern of the clinical response, there is the need to understand the pharmacodynamic (PD) contribution to bacterial killing i.e. how different sub-populations of *Mtb* respond to specific drug treatment (e.g. refs 10–12), and pharmacokinetic (PK) contribution to killing dynamics i.e. the ability of a drug to reach *Mtb* sub-populations residing in different cells, matrices and tissues (e.g. refs 10, 13, 14).

Critically, the PK and/or PD characteristics of a drug(s) that are most relevant in predicting the clinical response of an anti-tubercular drug/drug combinations remain to be defined and validated. Currently, several pre-clinical animal and *in vitro* efficacy models are being evaluated to determine their value in forecasting clinical outcomes^2, 3^.

It has long been recognized that intracellular *Mtb* growth and survival within macrophages plays a major role in TB pathogenesis^15^. Intracellular survival necessitates metabolic and physiological adaptations relative to extracellular growth. Significantly, *Mtb* that are genetically manipulated to lack genes required for survival within macrophages fail to establish pathogenicity in TB animal models^16–19^. Consequently, it is clear that demonstration of efficacy against intracellular *Mtb* must be a critical PD feature for any anti-tubercular drug under development^20^.

The importance of determining the PD response of intracellular *Mtb* is underscored by studies that demonstrate differential susceptibility to existing and novel anti-tubercular drugs and inhibitors against intracellular *versus* extracellular *Mtb* (e.g. refs 21–23). However, to date, studies assessing intracellular *Mtb* sensitivity to drugs do not allow for extrapolation and prediction of the clinical potential of the developing therapy. To address this deficiency, here we describe a new high-content imaging-based, PK-PD approach that utilizes concentration and time-dependent phenotypic data of intracellular *Mtb* residing within macrophages exposed to first-line TB drugs. We demonstrate that the use of intracellular *Mtb*-derived PD data, alongside conventional extracellular *Mtb* PD data, results in the generation of superior clinical predictions of TB treatment duration with biphasic responses akin to those described previously in the clinic. In addition, we show that our model can be used to simulate new therapeutic regimens such as the use of higher doses of RIF (35 mg/kg) to predict the outcomes of clinical studies. We anticipate that our described high-content imaging platform and PK-PD approach will be an invaluable tool that can correctly identify new combination therapies that can genuinely reduce TB treatment duration in the clinic.

## Results

### Growth rate of *Mtb* within macrophages is significantly reduced compared to planktonic *Mtb* grown in liquid culture

**Figure 1:**
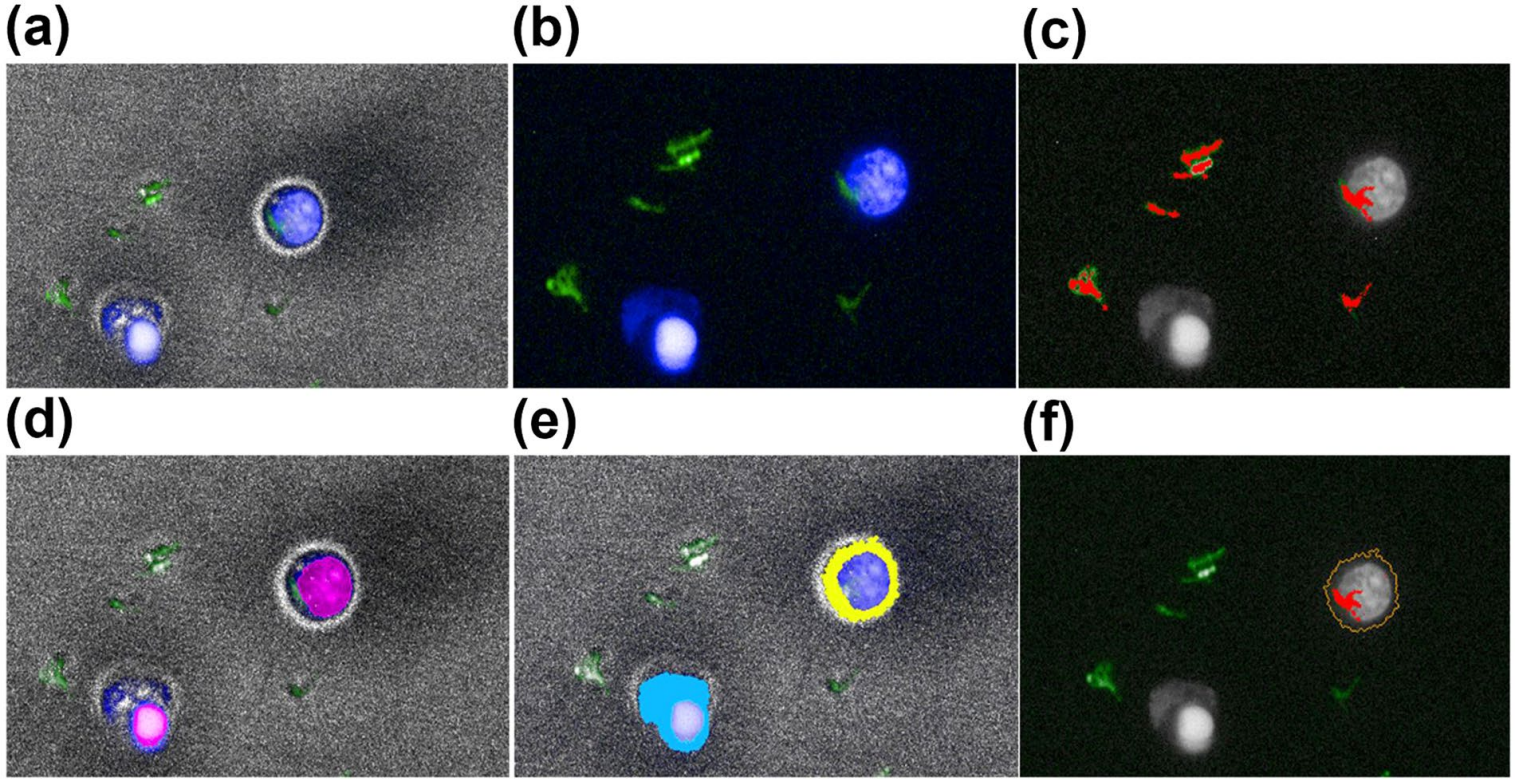
Automated work flow of image analysis in Harmony. THP-1 macrophages infected with GFP-H37Rv were imaged on the Operetta platform described in the Methods section. The images were subsequently analysed with Harmony software in the following work flow (**a**) Image capture from one field of view displaying brightfield (grey), Hoechst (blue), and GFP-H37Rv (green). (**b**) Image without brightfield, displaying Hoechst (blue) and GFP-H37Rv (green). (**c**) Identification of extracellular GFP-H37Rv, detected *M. tuberculosis* are highlighted in red, the nucleus of the macrophages is in grey. (**d**) Identification of the nucleus of macrophages, detected nuclei (pink). (**e**) Cytoplasm detection, cytoplasm in blue and yellow. (**f**) *M. tuberculosis* infected macrophage (outline in orange), intracellular *M. tuberculosis* highlighted in red.

The described optimized high-content imaging platform permitted spatial measurement of H37Rv-GFP *Mtb* residing within macrophages (Fig. 1). Longitudinal data of intracellular H37Rv-GFP *Mtb* growth over 5 days were analyzed using an exponential, capacity saturated model (see Methods) and the growth rate was calculated as 0.033 h^−1^ (doubling time 21 h, Table 1 and Supplementary Fig. 1). These data are delineated from *Mtb* growing in liquid culture, (doubling time determined to be approximately 9 h) based on colony count data generated in house (Supplementary Fig. 1 and ref. 24) which agrees with data reported elsewhere^25^.

**Table 1:** Comparison of growth and pharmacodynamic killing kinetics for planktonic (extracellular liquid culture) and intracellular (macrophage) *M. tuberculosis*.

Treatment	Parameter	Macrophage (intracellular *Mtb*)	Liquid* (extracellular *Mtb*)
Control	*Kg*_*max*_ (h^−1^) (D.Time)	0.033 (21.0 h)	0.0769–0.045 (9.0 h)
Rifampicin	E_max_ (h^−1^)	0.055	0.178
	EC_50_ (ng/mL)	18.4	5.60
Ethambutol	E_max_ (h^−1^)	0.053	0.142
	EC_50_ (ng/mL)	79.5	264
Isoniazid	E_max_ (h^−1^)	0.041	0.710, 0.055
	EC_50_ (ng/mL)	32.1	790
Pyrazinamide	E_max_ (h^−1^)	0.043	NA
	EC_50_ (ng/mL)	45.5	NA

*Kg*_*max*_ represents the maximal growth rate, *E*_*max*_ represents the maximal possible kill rate and EC_50_ the concentration required to achieve 50% of the *E*_*max*_ rate of kill. Parameters were derived from data generated from multiple independent experiments (n ≥ 3) performed in triplicate. NA, Not applicable. *For comparison, extracellular PD parameters based on in house and published values^24, 25^.

### Intracellular *Mtb* residing within macrophages are killed more slowly by first line drugs

The optimized high-content imaging platform was used to measure the pharmacodynamic response of intracellular *Mtb* within macrophages to first line TB drugs at concentrations spanning the pharmacological range over an exposure period up to 120 h. Optimal assay performance was assessed and confirmed by a mean *Z*′-factor of 0.67 for the complete data set.

**Figure 2:**
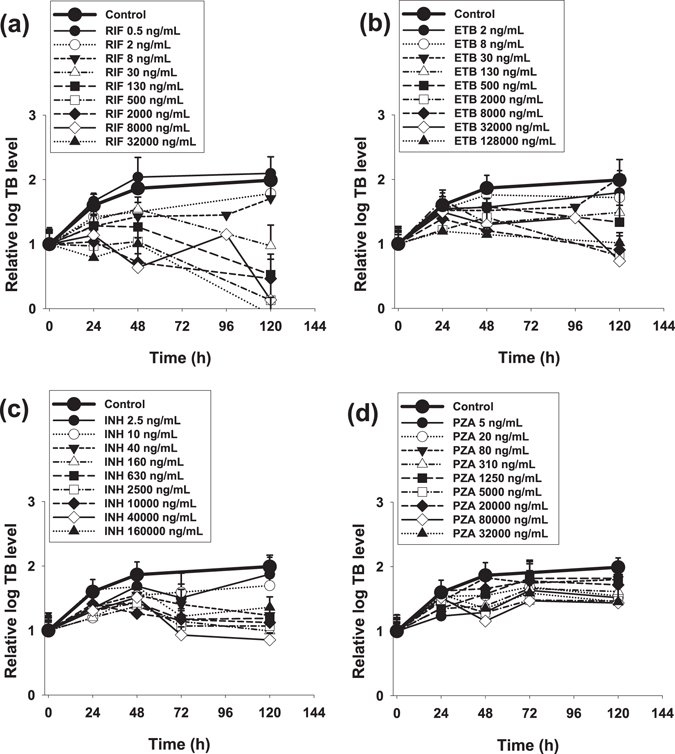
Intracellular (macrophage) *M. tuberculosis* time-dependent kill. Panels display time-kill profiles of RIF (**a**), ETB (**b**), INH (**c**), and PZA (**d**). Data is mean ± S.D derived from multiple independent experiments (*n* ≥ 3) performed in triplicate.

RIF, INH, ETB and PZA exhibited time- and concentration-dependent anti-tubercular activity against intracellular *Mtb* (Fig. 2). The kill dynamics for each drug was modelled as described in eq. 2 and the resultant kinetic parameters *E*_*max*_ (Maximum kill rate of a drug) and EC_50_ (concentration required to achieve half of this maximal kill rate) are presented in Table 1. In terms of intracellular *Mtb* killing dynamics, RIF ranked as the most effective out of all four drugs exhibiting an *E*_*max*_ value of 0.055 h^−1^ and an EC_50_ value of 18.4 ng/mL, followed by ETB, PZA and then INH. The raw (Fig. 2) and simulated (Supplementary Fig. 2) time- and concentration-dependent intracellular *Mtb* killing kinetics revealed that maximal killing was achieved for all drugs after an initial lag period of 20–48 h.

Comparison of intracellular and extracellular killing dynamics for all drugs show that intracellular *Mtb* residing inside macrophages exhibited much slower *E*_*max*_ values compared to kill rates shown previously in liquid cultures^24, 25^ and parameterised in Table 1.

**Figure 3:**
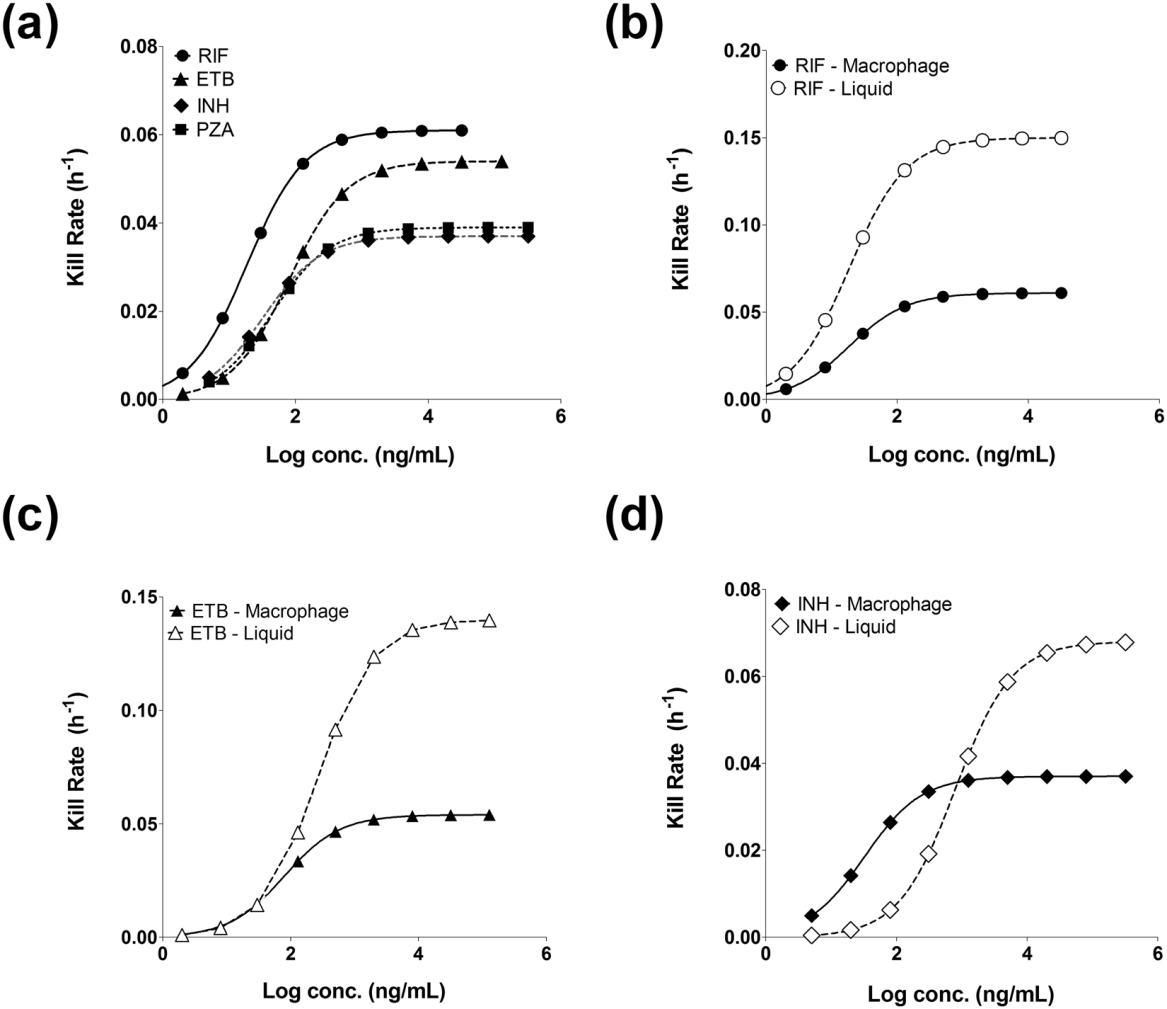
Intracellular (macrophage) and extracellular *M. tuberculosis* time-kill relationships. (**a**) Simulated concentration–kill rate relationship based on parameters generated from analysing intracellular Mtb kill curves for RIF (black circles), ETB (black triangles), INH (black diamonds) and PZA (black squares). (**b**) shows the concentration–kill relationship for RIF intracellular (black circles) and extracellular (open circles). (**c**) show the concentration–kill relationship of ETB intracellularly (black triangles) and extracellularly (open triangles) and (**d**) for the concentration–kill relationship of INH intracellularly (black diamonds) and extracellularly (open diamonds).

To facilitate the comparison, concentration-kill rate relationship plots were performed for each drug (Fig. 3). A comparison of the concentration-intracellular kill rate relationship for all 4 drugs reveals the superiority of RIF over the other drugs, displaying the highest achievable *E*_*max*_ and a low EC_50_ (Fig. 3a). Plots comparing concentration-kill rate relationships for extracellular^25^ versus intracellular *Mtb*, show that despite broadly comparable EC_50_ levels, RIF, ETB and INH all display accelerated kill rates in the liquid assay compared to kill rates for intracellular *Mtb* (Fig. 3b–d).

### PK-PD modelling using macrophage intracellular *Mtb* kill rates is predictive of treatment duration

To determine whether the PD data acquired from the intracellular macrophage high content platform has value in forecasting clinical TB treatment scenarios, Monte-Carlo PK-PD simulations for RIF, ETB, INH and PZA were conducted based on 1,000 theoretical subjects. Using published clinical PK parameters^26–31^, simulations were performed for different treatment regimens using either intracellular or extracellular *Mtb* PD data, or a combination of both.

**Figure 4:**
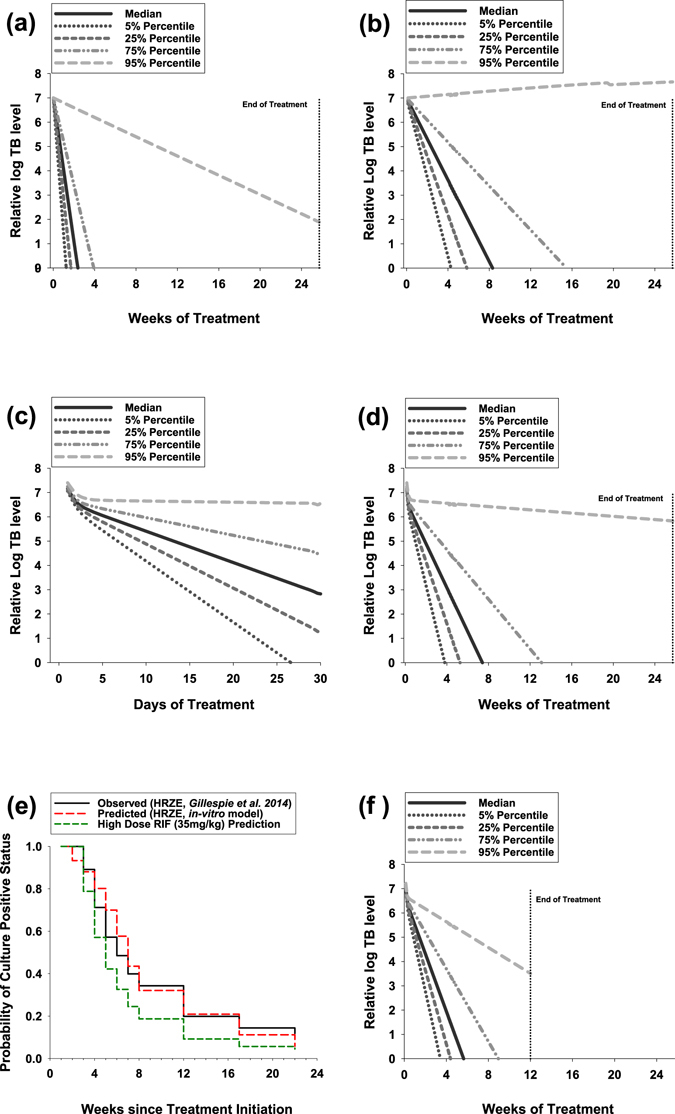
PK-PD Monte-Carlo simulations of clinical *Mtb* response based on concentration-kill rate dynamics derived from *in vitro* models of intracellular and extracellular *Mtb*. (**a**) Predicted dynamics of total TB in patients receiving HRZE combination for 6 months based on extracellular kill rates only. (**b**) Predicted dynamics of total TB in patients receiving HRZE combination for 6 months based on intracellular kill rates only. (**c**) Predicted dynamics of total TB in patients receiving HRZE combination for 6 months based on the assumption that extracellular TB constitutes 95% and intracellular 5% of total TB (1-month view). (**d**) Predicted dynamics of total TB in patients receiving HRZE combination for 6 months based on the assumption that extracellular TB constitutes 95% and intracellular 5% of total TB (6-month view). (**e**) Probability of sputum sample conversion to *Mtb* culture-positive status over time as observed in TB patients receiving standard HRZE treatment in a previous clinical study^7^ (solid black line) compared to the predicted probabilities over time using our novel PK-PD model (dashed red line). Dashed green line shows our PK-PD prediction when using a standard HRZE regimen but with an elevated dose of RIF (35 mg/kg). The comparison assumes that the limit of detection for positive culture conversion is 10 CFU/mL when using Löwenstein–Jensen medium culture assays^67^ which have been implemented in the comparator clinical study. (**f**) Predicted dynamics of total TB in patients receiving HRZE with a high dose of RIF (35 mg/kg) for 3 months on the assumption that extracellular TB constitutes 95% and intracellular 5% of total TB (6-month view).

A simulation of the recommended WHO treatment regimen of an intensive 8 week treatment phase of RIF, ETB, INH and PZA followed by a 16 week continuation phase of RIF, INH using PD parameters taken from extracellular (liquid culture) *Mtb* data, reveal that median clearance time to achieve a 10^7^ *Mtb*/ml log drop would take 16 days (Fig. 4a). This predicted rapid clearance contrasts with the simulations using intracellular *Mtb* PD parameters (Table 1 and Fig. 3) which predict a median clearance time of 56 days to achieve the same reduction of *Mtb* as predicted based on liquid culture (Fig. 4b). Significantly, simulations that combine both extracellular and intracellular *Mtb* PD parameters, result in a biphasic response for *Mtb* clearance, the intersection of which is dependent on the starting proportions of the two populations (Fig. 4c). The majority of *Mtb* in advanced disease states are found to be extracellular^32, 33^. Simulations of a standard 6 month treatment as per WHO recommendations, assuming that 95% of Mtb is extracellular and the rest 5% intracellular, results in 83% of patients achieving cure whilst the 17% remaining would be predicted to fail treatment (Fig. 4d). Notably, it is the killing dynamics of the intracellular *Mtb* population that predominantly determine the treatment duration. Sensitivity analysis across a wide range of intracellular:extracellular ratio fractions (1%:99%–99%:1%) results in minimal variation (<8%) in total population achieving cure at the end of standard therapy whilst variation in median time to achieve cure changes by no more than 13 days (22%) regardless of assumed initial intracellular/extracellular ratio at the beginning of the simulation (Supplementary Fig. 3). We conducted a further comparison of our simulated standard 6 month treatment with clinical data obtained from the control arm of a recent Phase 3 trial (REMoxTB^7^). Kaplan-Meier estimates showing the time until conversion to culture-negative status, show that our simulations using growth and kill curves from 95:5% extracellular: intracellular *Mtb*-based PK-PD modelling compare very favourably with the clinical scenario (Fig. 4e).

Using our models, it is possible to make predictions of the clinical outcomes of proposed trials using the drugs we have investigated such as proposed high dose rifampicin^34, 35^. Simulations using high dose RIF (35 mg/kg) exposures again show a biphasic response due to differential killing dynamics of extracellular and intracellular *Mtb* (Fig. 4f). Significantly, assuming a 95:5%, extracellular:intracellular ratio of *Mtb*, simulations predict that only 12 weeks (~3 months) of a high dose RIF-containing treatment should deliver a clinical outcome equivalent to a 6 month HRZE standard course (87% cure and 13% failure rate, Fig. 4f). A predicted reduced time until conversion to culture-negative status for high dose RIF compared to standard treatment is further illustrated in Kaplan-Meier simulations (Fig. 4e).

## Discussion

Survival of *Mtb* within macrophages is a critical aspect of TB pathogenicity^15^. TB drug discovery efforts that have failed to simulate physiological growth conditions have resulted in the identification of false positive hits in drug screening and discovery programmes that have translated poorly to *in vivo* models (and man) and that are therefore not developable^3, 36^. The intracellular macrophage environment offers the ability to measure *Mtb* drug dynamics within an environment that better reflects the common niche of this pathogen. Reflecting the nutritionally-constrained environment of the intracellular macrophage milieu^37^, the population growth of intracellular *Mtb* residing within macrophages was much slower (~21 h doubling time, Table 1 and Supplementary Fig. 1) compared with *Mtb* grown in stirred liquid culture which has a doubling time of approximately 9 h^24, 25^ (Table 1 and Supplementary Fig. 1).

Drug susceptibility of *Mtb* residing within macrophages has previously been shown to correlate poorly with that of extracellular *Mtb*. This has been explained by invoking selective inhibition of pathways essential for intracellular metabolism^21, 23^ or poor permeability/altered drug transport^38^ into these intracellular compartments. Our imaging data, measuring the intracellular *Mtb* response to first-line TB drugs, clearly demonstrates altered drug dynamics compared to extracellular *Mtb* (Table 1, Figs 2 and 3). Most notably, the maximum rate of *Mtb* elimination (*E*_max_) that is achievable for RIF, ETB and INH is dramatically reduced compared to extracellularly grown *Mtb* (Table 1, Figs 2 and 3). Significantly, the inability to increase the *E*_max_ using super-pharmacological concentrations of drug, suggests that this phenomenon is not solely linked to intracellular drug availability which has been linked to altered RIF susceptibility when measured using static assays^38^. Instead, we hypothesise that the kill rate (*E*_max_) of RIF, INH and ETB (a comparison of PZA cannot be made using the standard liquid culture conditions used here) is intimately linked to the inherent growth rate of the intracellular *Mtb* residing within the nutritionally-constrained environment of the macrophage (the same logic would apply to *Mtb* in any intracellular/compartmental niche that constrains growth dynamics). A link between growth-rate and kill rate for antibiotics that target the replication machinery of the cell has been described for many years (e.g. refs 39–41). The mechanisms underpinning growth-rate dependent killing of some antibiotics are beginning to be understood. Antibiotics targeting the ribosome for example, growth-rate-dependent killing is explained by the growth rate-dependent partitioning of the cell’s translational resources between production of new ribosomes and production of other proteins^40, 42, 43^. Single-cell studies have also recently confirmed that phenotypic antibiotic tolerance, often described as persistence, can be demonstrated in actively growing bacteria^44^, including *Mycobacterium* species^45^ and is not therefore wholly dependent on rare stochastic non-dividing (also known as dormant) populations^46^.

The significance of reduced kill rate (*E*_*max*_) values for RIF, INH and ETB in intracellular *Mtb* experiments relative to kill rate (*E*_*max*_) values derived from liquid culture experiments of extracellular *Mtb*, is that PK-PD predictions of clinical scenarios can be radically skewed from clinical reality. This is illustrated in the PK-PD simulations performed using extracellular (Fig. 4a) and intracellular (Fig. 4b) *Mtb* drug dynamic parameters, resulting in dramatically different times to culture conversion. The majority of *Mtb* in advance disease state are found to be extracellular^32, 33^, assuming a ratio of 95% extracellular to 5% intracellular *Mtb* following treatment results in biphasic TB elimination profiles (Fig. 4c) that are similar to the treatment responses reported clinically following short-course regimens^47, 48^. It is noteworthy that treatment response in terms of both duration and cure (%) displayed only minimal variation in sensitivity analyses performed across a wide range of intracellular:extracellular ratio fractions (1%:99%–99%:1%, Supplementary Fig. 3).

Simulations of a standard 6 month treatment as per WHO recommendations, assuming that 95% of TB is extracellular and the rest 5% intracellular, results in 83% of patients achieving cure whilst the 17% remaining would be predicted to fail treatment (Fig. 4d). This agrees with literature data relating to relapse rates after standard 6 month TB treatment^49^. Simulations of standard WHO treatment duration lasting 2 months are predicted to result in a 50% cure rate, this is again in agreement with clinical studies^50, 51^. Moreover, the simulations are consistent with clinical observations showing a predominant role for INH during the initial fast *Mtb* elimination phase (INH was measured to have the fastest *E*_max_ for extracellular *Mtb*, Table 1) whilst RIF has the predominant role during the second slower elimination phase (RIF was measured to have the fastest *E*_max_ for intracellular *Mtb*, Table 1)^52^.

The described high-content imaging platform makes possible the dynamic measurement of intracellular Mtb in response to any drug. By linking the intracellular kill kinetics with drug exposure, and in this regard we underline the importance of acquiring accurate drug measurements in relevant compartments/tissues (e.g. ref. 13), it is possible to simulate clinical scenarios that can be validated against actual trial data. Here, we described this approach for high dose RIF trials (35 mg/kg) which are currently under clinical evaluation (e.g. refs 34, 35). Strikingly, simulations using PD data from intracellular *Mtb* exposed to high RIF concentrations (Fig. 2a) predict that clinical outcome equivalent to a 6 month HRZE standard course (83% cure and 17% failure rate, Fig. 4e) would occur within a significantly shorter time period (12 weeks) of a high dose RIF-containing treatment, reducing treatment therefore by approximately a half of the usual length. Our simulations are consistent with clinical data from ﻿Phase II studies in which rate increases in the dose of RIF result in an accelerate rate of decline in bacterial load^34, 53–56^.

*Mtb* within macrophages play a critical role in the life history of TB infection. The dynamic drug response of intracellular *Mtb* residing inside macrophages is dramatically different to that of extracellular *Mtb*. In essence, for drugs affecting the replication machinery, maximum kill kinetics are intimately linked with compartmentalized growth dynamics. The critical importance of this observation is that the dynamic intracellular *Mtb* drug response (compared to extracellular killing) provides the most predictive estimates of clinical treatment response. The described dynamic high-content imaging platform offers a new tool to aid decision-making and is recommended for incorporation as an essential assay for compound progression within the TCP (target candidate profiles) of future TB drug discovery programmes aiming to identify therapies that reduce treatment duration.

## Methods

### Chemical Compounds

The compounds Isonicotinic Acid Hydrazide (Isoniazid, INH), Rifampicin (RIF), Ethambutol Dihydrochloride (ETB), Pyrazinoic Acid Amide (Pyrazinamide, PZA) were purchased from Sigma. All compounds, except Pyrazinamide, which was made up in media, were made up in DMSO (Sigma). Final DMSO concentration was adjusted and used at 0.1% in all test conditions.

### Mycobacterial Strains and *Mtb* Growth

*Mtb* H37Rv expressing a green fluorescent protein (H37Rv-GFP) was used in this study. The recombinant H37Rv-GFP contains an integrative plasmid (pENVY1) carrying the Green Fluorescent Protein (*gfp*) gene constitutively expressed from a HSP60 promoter (a kind gift from Professor T. Parish). H37Rv-GFP was pre-cultured aerobically at 37 °C in Middlebrook 7H9 broth (Difco) supplemented with 0.05% (v/v) Tween 80 (Sigma), 0.2% (v/v) glycerol, 10% oleic acid-albumin-dextrose-catalase (OADC) (hereafter called 7H9) and 50 µg/ml hygromycin (Sigma) for 14 days.

### Extracellular *Mtb* time-kill studies

Mid-log phase *Mtb* H37Rv was diluted to 1 × 10^7^ CFU/mL in 7H9. Aliquots of 1.5 mL of bacterial culture with magnetic stirrers was incubated aerobically at 37 °C in the presence or absence of drug. At three time points per day for up to 7 days, optical density (600 nm) was measured (beginning from day 0). In experiments in which colony forming units (CFUs) were measured, aliquots of culture were withdrawn at periodic intervals and pelleted material washed in drug-free Middlebrook 7H9 media before CFU/mL determination by colony counting on solid growth media containing Middlebrook 7H11 agar plates supplemented with 10% oleic acid–albumin–dextrose–catalase solution (Becton Dickinson), 0.2% (v/v) glycerol and 0.05% (v/v) Tween 80.

### Macrophage Infection Assay in 96-Well Plates, High-content Image Acquisition and Analysis

THP-1 cells were routinely cultured in RPMI 1640 with L-Glutamine and NaHCO_3_ (Gibco) supplemented with 10% heat-inactivated foetal bovine serum (FBS; Gibco), at 37 °C, 5% CO_2_. For the infection assay THP-1 cells were differentiated in 96-well Matriplate plates (MGB096-1-2-LG-L Black 0.17 low glass; Brooks). THP-1 cells were seeded at 5 × 10^5^ cells per well and differentiated for 72 h in RPMI 1640 with L-Glutamine and NaHCO_3_ supplemented with 10% heat-inactivated FBS and 100 ng/ml phorbol 12-myristate 13-acetate (PMA; Sigma) at 37 °C, 5% CO_2_. The medium was removed after 72 h and THP-1 cells incubated for a further 24 h in RPMI 1640 with L-Glutamine and NaHCO_3_ supplemented with 10% FBS at 37 °C, 5% CO_2_. Differentiated THP-1 cells were infected with H37Rv-GFP in suspension at a multiplicity of infection (MOI) of 1:5 in RPMI 1640 with L-Glutamine and NaHCO_3_ supplemented with 10% heat-inactivated FBS for 24 h at 37 °C. After 24 h the cells were washed and the drugs at the required concentrations added. Infected cells were incubated for up to 5 days at 37 °C, 5% CO_2_. At each time point, plates were fixed with 5% paraformaldehyde (PFA) (Sigma) for 2 h before staining. Macrophages were stained with Hoechst 3442 (Invitrogen) at 2 µg/mL in PBS for 30 min at 37 °C. Image acquisition was performed with an Operetta (PerkinElmer) using a 60x High NA objective. H37Rv-GFP were detected using 460/490 nm excitation coupled with a 500/550 nm detection filter and Hoechst 3442 labeled cells were detected using excitation at 360/400 nm laser coupled with a 410/480 nm detection filter. Fourteen fields and a Z stack of 6 intervals over 0–6 µm were recorded for each plate well. Each image was processed using Harmony 3.5.1 analysis software (PerkinElmer). Briefly, the cell nucleus was segmented using detection of Hoechst 3442. From this the cell area was defined again using Hoechst 3442 (due to cellular RNA staining). Within each macrophage the spot finding algorithm was utilised to detect the area of H37Rv-GFP in each cell. The output parameter deduced from the images was the bacterial load, which refers to the total surface area of all the green objects that reside within the macrophages, as a ratio of total cell area. Data were generated from multiple independent experiments (n ≥ 3) performed in triplicate. Assay performance was measured using *Z*′-factor determination^57^.

### Pharmacodynamic data analysis

The growth dynamics of *Mtb* were calculated according to an exponential, capacity saturated model which takes into account the saturation of growth that was observed at day 5 with control experiments. The growth rate was calculated according to Eq. 1 taken from^58^:

1 $$\frac{dMtb}{dt}=Mtb\cdot K{g}_{max}\cdot (1-\frac{Mtb}{POPMAX})$$

where *Mtb* represents the total *Mtb* count in the well at any given time (*t*), *Kg*_*max*_ represents the maximal growth rate per unit time and POPMAX represents the maximum capacity of the *Mtb* population in a well, in other words the maximum limit of *Mtb* number per well.

A model that calculates the kill dynamics of drugs over multiple time points was developed based on previous work^58^, in contrast to calculating fixed time point MIC or IC_50_ values. The kill dynamics for each drug in the assay were modelled simultaneously over 5 days with all the tested concentrations (a gradient of 12 concentrations per drug ranging from 0.5 ng/mL to 32 µg/mL). We use the term ‘kill rate’ to describe the rate of reduction of *Mtb* growth per unit time, e.g. when the kill rate is equal to the growth rate, the number of *Mtb* will remain constant and when the kill rate exceeds the growth rate, the number of *Mtb* will decline over time, and vice versa. The model calculates the most likely values of *E*_*max*_ (Maximum possible kill rate of a drug per unit time) and *C*_*50*_ (concentration required to achieve half of this maximal kill rate) based on all the available data from all the tested drug concentrations according to Eq. 2:

2 $$\frac{dMtb}{dt}=Mtb\cdot K{g}_{max}\cdot (1-\frac{Mtb}{POPMAX})-Mtb\cdot \,\frac{{E}_{max}\cdot conc.}{{C}_{50}+conc.}$$

Using Eq. 2 the model calculates the maximal kill rate of each drug per unit time as well as the *C*_*50*_ value that is required to achieve half of that rate.

For extracellular Mtb growth and kill dynamics, we used a combination of in-house data of *Mtb* grown (planktonic) in liquid media as well as previously published kill dynamic data of first-line drugs published by our laboratory^24^ and that of other groups e.g. ref. 25 using the same model to allow for intracellular-extracellular dynamics comparisons.

### Monte-Carlo Simulations

The growth and kill parameters estimated from the intracellular and extracellular assays were used to predict the PK-PD relationship in a clinical context. The PK parameters of all four drugs were taken from the literature^26, 27, 30, 31, 59–66^. The PK-PD model linked the PK to the PD component by using the simulated dynamic drug concentrations in a patient to represent ‘conc’. In Eq. 2. When combining multiple drugs it was assumed that the rate of kill is equal to that of the drug that achieves the higher kill rate based on its concentration and kill rate (based on the argument that you can only kill once and there is no underlying synergy/antagonism between the drugs). This was achieved by using IF statements in the Pmetrics model file that are applied at each time step. The simulation was then run assuming that all four drugs are administered concomitantly once daily for a period of 8 weeks followed by RIF and INH for a further period of 18 weeks, as per standard WHO treatment recommendations. The simulation is aimed at identifying the time it takes to achieve a 7 log reduction of *Mtb*, as such a drop is correlated with a cure in clinical studies^48^. Variability was set at 30% for the PK parameters whilst the variability and limits of the PD parameters were set according to the modelling of the *in-vitro* data.

## Electronic supplementary material

Supplementary Figures
