# Supplementary material for: Pharmacokinetic-Pharmacodynamic modelling of intracellular Mycobacterium tuberculosis growth and kill rates is predictive of clinical treatment duration
Source: Sci Rep. 2017 Mar 29;7:502. doi: 10.1038/s41598-017-00529-6 (PMC5428680; doi:10.1038/s41598-017-00529-6)
Supplement: Supplementary file 1 — Supplementary Figures [file 41598_2017_529_MOESM1_ESM.pdf]

Supplementary Information

Pharmacokinetic-Pharmacodynamic modelling of intracellular *Mycobacterium tuberculosis* growth and kill rates is predictive of clinical treatment duration

Ghaith Aljayyoussi, Victoria A Jenkins, Raman Sharma, Alison Ardrey, Samantha Donnellan, Stephen A. Ward, Giancarlo A. Biagini*

Supplementary Figure Legends

Supplementary Figure 1. Growth rate of intracellular (macrophage, black circles) and extracellular (planktonic liquid culture, open circles) *M. tuberculosis*. Growth rates showing data obtained in house for intracellular and extracellular *Mtb* over 5-7 days.

Supplementary Figure 2. Intracellular (macrophage) *M. tuberculosis* time-kill kinetics. Simulations of time kill dynamics of (a) RIF, (b) ETB, (c) INH, and (d) PZA. The simulations are based on the final model parameters and overlaid with data ± S.D. for selected concentrations (selected from 9 concentrations ranges). Simulations were derived from data generated from multiple independent experiments ($n \geq 3$) performed in triplicate. All data were normalised to have the same starting point and TB levels are expressed relatively

Supplementary Figure 3. Sensitivity analysis to determine the influence of intracellular/extracellular *Mtb* ratio upon treatment simulations. Sensitivity analysis probing the effect of assumed initial intracellular fraction of *Mtb* upon the overall outcome of therapy displayed as % patients who are predicted to achieve cure at the end of a standard therapy regime (black line, left y axis) and as the median time it takes to achieve cure in 50% of the population (dashed grey line, right y axis). Vertical black dashed line marks the assumed initial percentage of *Mtb* in the simulations presented in the current work.

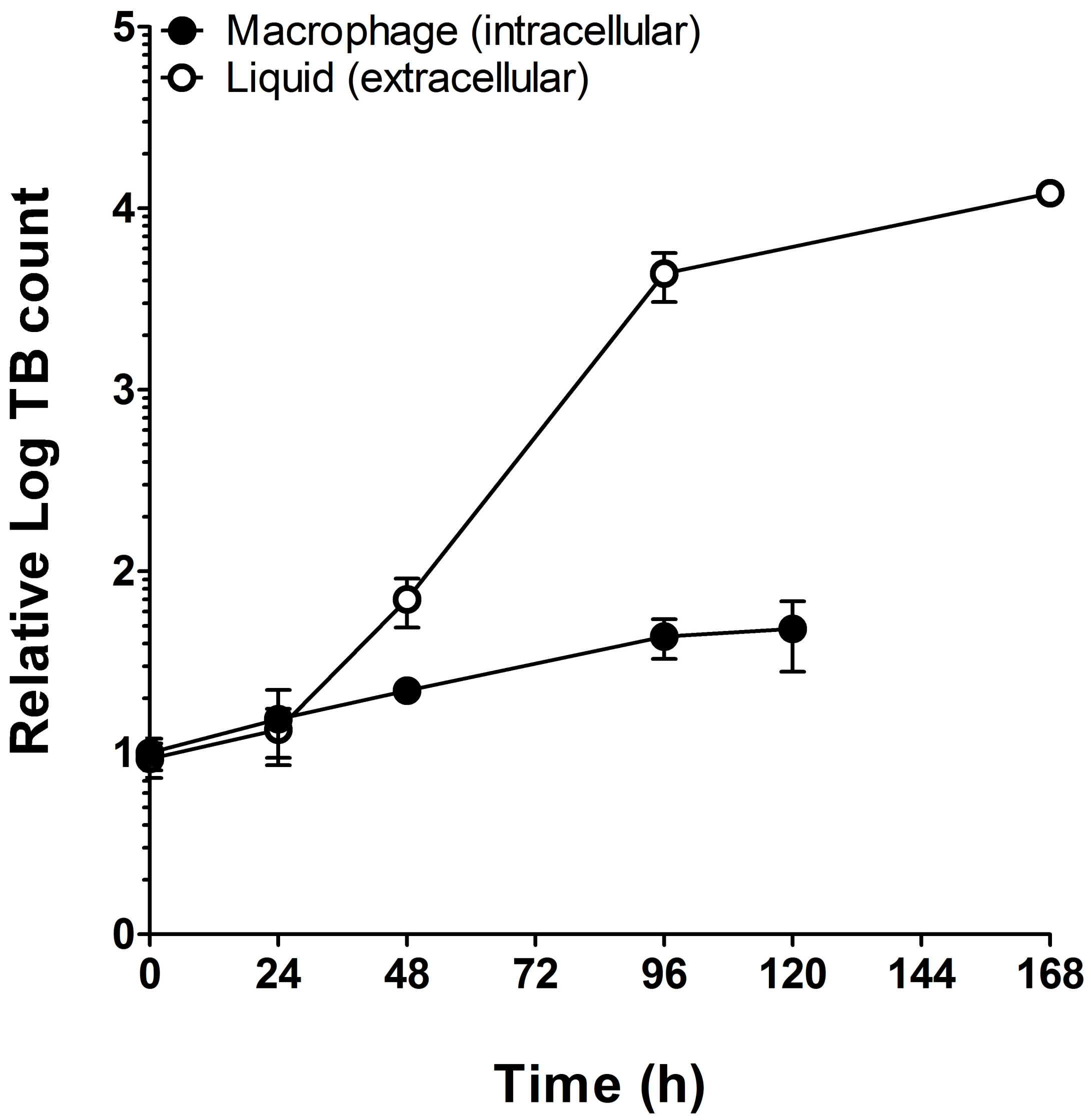

Supplementary Figure 1

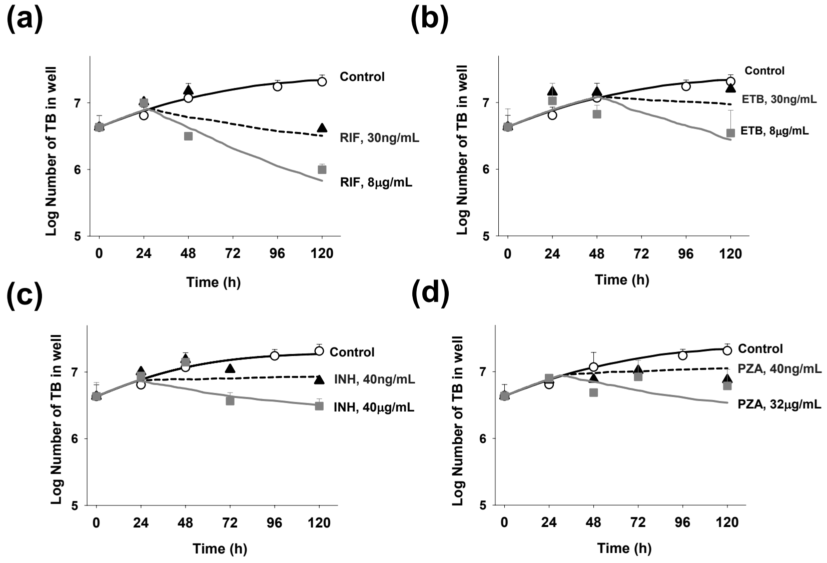

Supplementary Figure 2

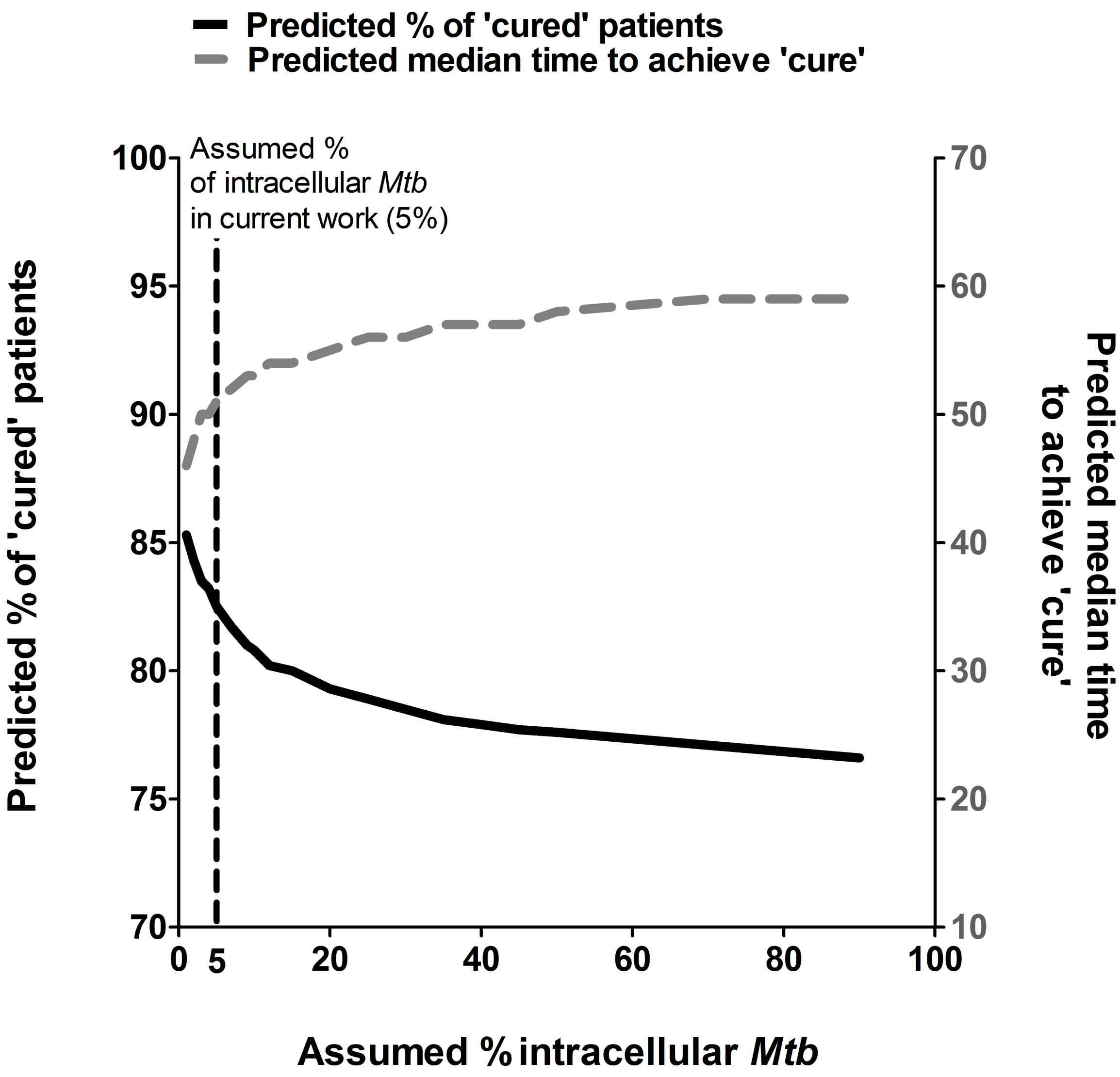

Supplementary Figure 3
